# Comparison of cardiovascular response to sinusoidal and constant lower body negative pressure with reference to very mild whole-body heating

**DOI:** 10.1186/1880-6805-31-30

**Published:** 2012-11-24

**Authors:** Keita Ishibashi, Takafumi Maeda, Shigekazu Higuchi, Koichi Iwanaga, Akira Yasukouchi

**Affiliations:** 1 Department of Design Science, Graduate School of Engineering, Chiba University, 1-33, Yayoi, Inage, Chiba, 263-8522, Japan; 2 Faculty of Engineering, Hokkaido University, Sapporo, Japan; 3 Faculty of Design, Kyushu University, Fukuoka, Japan

**Keywords:** Hemodynamics, Lower body negative pressure, Hyperthermia, Fourier analysis, Autonomic nervous system, Chronobiology phenomena

## Abstract

**Background:**

The purpose of the present study was to compare sinusoidal *versus* constant lower body negative pressure (LBNP) with reference to very mild whole-body heating. Sinusoidal LBNP has a periodic load component (PLC) and a constant load component (CLC) of orthostatic stress, whereas constant LBNP has only a CLC. We tested two sinusoidal patterns (30-s and 180-s periods with 25 mmHg amplitude) of LBNP and a constant LBNP with −25 mmHg in 12 adult male subjects.

**Results:**

Although the CLC of all three LBNP conditions were configured with −25 mmHg, the mean arterial pressure (MAP) results showed a significantly large decrease from baseline in the 30-s period condition (*P* <0.01). In contrast, the other cardiovascular indices (heart rate (HR), stroke volume (SV), cardiac output (CO), basal thoracic impedance (Z_0_), total peripheral resistance (TPR), the natural logarithmic of the HF component (lnHF), and LF/HF (ln(LF/HF))) of heart rate variability (HRV) showed relatively small variations from baseline in the 30-s period condition (*P* <0.01). The result of the gain and phase of transfer function at the sinusoidal period of LBNP showed that the very mild whole-body heating augmented the orthostatic responses.

**Conclusion:**

These results revealed that the effect of the CLC of LBNP on cardiovascular adjustability was attenuated by the addition of the PLC to LBNP. Based on the results of suppressed HRV response from baseline in the 30-s period condition, we suggest that the attenuation may be caused by the suppression of the vagal responsiveness to LBNP.

## Background

Lower body negative pressure (LBNP) is used as a perturbation to the cardiovascular system and has been applied to simulate the gravitational stress of orthostatic blood shift in humans [1,2]. Orthostatic faint is one of the non-adaptive responses to the gravitational stress in humans, and that has not been described in apes [3]. The metabolic demand of a human’s large brain needs a large proportion of cardiac output (CO) to be pumped upward and the relative long leg causes the large amount of blood pooling in the leg in the orthostatic stress [3-5]. It is presumed that the combination of these anatomical features of large brain and long leg in human evolution causes orthostatic difficulty.

The transient phenomenon is one of the features of orthostatic faint [6,7]. Oscillatory LBNP methods have been used in several studies to investigate the transient characteristics of the orthostatic response with high repeatability [8-10]. However, at present, sinusoidal LBNP methods are not major among oscillatory LBNP methods. Considering the spectral leakages of oscillatory LBNP [11], the waveform of LBNP should be a sinusoidal pattern during oscillatory LBNP. Although a few previous studies used sinusoidal LBNP as the perturbation stimulus [12,13], only Levenhagen *et al.* (1994) showed the result of an analysis of the actual gauge pressure of sinusoidal LBNP. They investigated the frequency characteristics of hemodynamic changes induced by sinusoidal LBNP in humans across a range of 10- to 250-s periods (that is, 0.1 to 0.004 Hz) [13]. They revealed that the cardiovascular adjustability to sinusoidal LBNP was maintained at a period slower than 50-s (that is, 0.02 Hz) oscillation.

**Figure 1:**
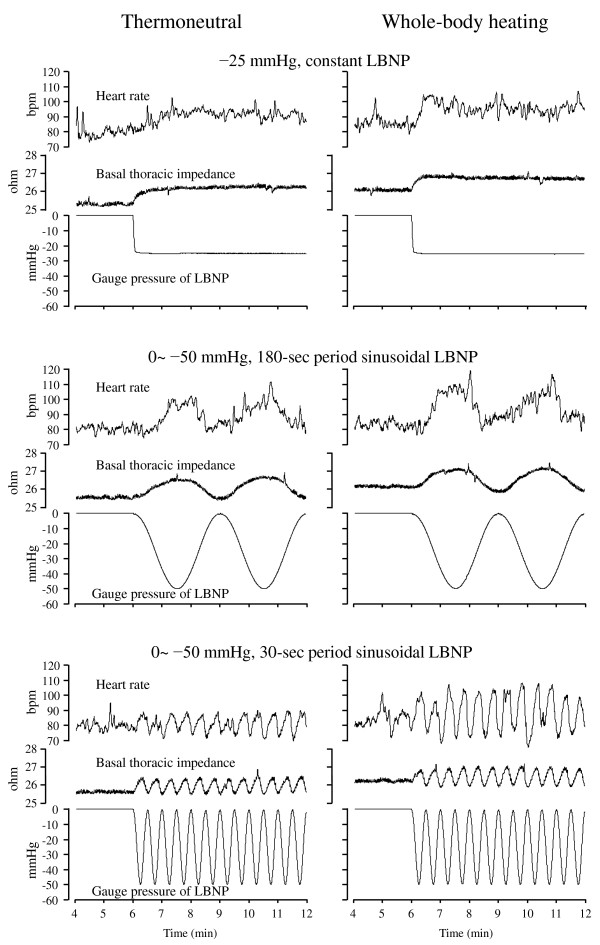
**Traces of heart rate (HR), basal thoracic impedance (Z_**0**_), and gauge pressure of LBNP from three conditions of LBNP (−25 mmHg constant LBNP, 30-s and 180-s period of sinusoidal LBNP) during thermoneutral and whole-body heating conditions in the same male subject.** The average value of LBNP (CLC) was configured with −25 mmHg in all conditions.

However, to our knowledge there is no previous study that directly compared sinusoidal LBNP and constant LBNP which is a conventional method in LBNP studies. Sinusoidal LBNP contains not only the periodic load component (PLC) but also the constant load component (CLC) of LBNP, whereas the constant LBNP contains only CLC (see Figure 1). CLC is the average load during LBNP, and that is a 0 Hz frequency (that is, infinite periodic) component in frequency analysis. The comparison of cardiovascular response to sinusoidal and constant LBNP under the same CLC condition could reveal the effect of PLC in sinusoidal LBNP.

Orthostatic cardiovascular regulation is compromised by heat stress [14,15]. Whole-body heating during LBNP causes peripheral vasodilation, decreases cerebral blood flow (CBF), and reduces orthostatic tolerance. Warm environments are described as one of the most common triggers for fainting episodes in the general population [16]. Although there is considerable inter-individual variation in orthostatic tolerance among normal subjects [7,17], evidence that fainting has a genetic basis is not very strong [18-20]. To elucidate the explanatory variables accounting for the large inter-individual variation in physiological responses, the relationship between reduced orthostatic tolerance in warm environments and the broad range of lifestyle habits such as habitual physical exercise and chronobiological rhythms of the subjects could also be investigated. Sedentary and evening type lifestyles differ with regard to their cardiovascular responses to stress [21,22]. In hindsight, although we used very mild whole-body heating in this study, the very mild hyperthermia effect on orthostatic cardiovascular response to sinusoidal and constant LBNP was investigated.

Accordingly, the first objective of the present study was to investigate the effects of CLC and PLC of LBNP with a comparison of the cardiovascular responses to sinusoidal LBNP and constant LBNP with reference to very mild whole-body heating. In light of the finding by Levenhagen *et al.* (1994) that a 50-s period was the boundary period of the AP regulation to sinusoidal LBNP, we tested two different periods (30 s and 180 s) of the PLC of LBNP. Our second objective was to investigate the relationship between the response to the LBNP and lifestyle habits of the subject, as an attempt to identify any explanatory variables for the large inter-individual variation in orthostatic tolerance.

## Methods

### Subjects

Twelve adult male subjects (age, 23.2 ± 0.3 years (standard error, or SE) (range, 21.8 to 25.4 years); height, 1.713 ± 0.015 m (SE) (range, 1.632 to 1.803 m); body mass, 59.9 ± 2.3 kg (SE) (range, 48.1 to 72.0 kg); body mass index (BMI; body mass/height^2^), 20.4 ± 0.7 kg/m^2^ (SE) (range, 15.8 to 24.2 kg/m^2^)) participated in the study. No subjects smoked or took medication regularly, and they refrained from alcohol consumption and heavy exercise for at least 24 h prior to participating in the study. The study was approved by the Research Ethics Committee of the Chiba University Faculty of Engineering (22–15). Written informed consent was obtained from all subjects before the start of the experiment.

### LBNP

We used the electronically controlled LBNP system that we devised and reported previously; the technical issues regarding the construction of the LBNP chamber and the electronic control of the LBNP are as described [23]. To improve the accuracy of the LBNP control, we installed a differential pressure gauge (PU-10kPa; Halstrup-Walcher, Kirchzarten, Germany) and an electronically controlled blower (VASF 1.50/1; Gebr Becker, Wuppertal, Germany) in the LBNP system. The controllable range of the pressure gauge of this LBNP system was from −0.60 to −60.00 mmHg with a 10-bit DA controller. Therefore, the air pressure from 0 (that is, sea level) to −0.60 mmHg was out of range of the controller.

Two sinusoidal patterns of LBNP and a constant LBNP were applied. The periods of sinusoidal curve were 30 s (0.033 Hz) and 180 s (0.0055 Hz). The range of the curve was configured using 0 to −50 mmHg. The constant LBNP level was set at −25 mmHg (Figure 1). The duration of the LBNP was 12 min. The baseline period was 6 min before LBNP. The average gauge pressures that were the PLC of all three LBNP conditions were set at −25 mmHg.

### Experimental protocol

**Figure 2:**
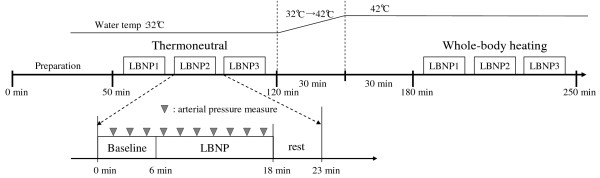
Experimental protocol.

The experimental protocol is shown in Figure 2. The experiments were carried out in a semi-dark thermoneutral room (temperature, 27.25°C ± 0.29°C (SE) (range, 26.62°C to 27.88°C); relative humidity, 52.2 ± 0.4% (SE) (range, 51.2% to 53.1%)). The subjects reported to the experimental room at 10:00 (*n* = 6), 12:00 (*n* = 1), or 16:00 (*n* = 5). Each subject’s preparation for the experiment started approximately 1 h prior to the start of the measurement: the subject emptied his bladder, had the electrode and thermistors attached. The subject exchanged his clothing for a long-sleeved T-shirt (KZ3002, Gunze, Kyoto, Japan) and long-johns (KZ3002, Gunze) to prevent low-temperature injury from the direct heating by the tube-line suit. Under the long-sleeved T-shirt, a neoprene skirt (KAKR002, Sandiline, Koper, Slovenia) for air-tight sealing was fitted around the subject’s iliac crest. Over his clothes and neoprene skirt, the subject wore a water-perfused tube-lined suit (shirt, 420–122; pants, 420–126, Med-Eng, Allen-Vanguard Corp., Ottawa, Canada) and was placed inside the LBNP chamber in the supine position. A saddle in the LBNP chamber provided perineal support and prevented caudal displacement of the subject. The subject was allowed to watch mildly stimulating movies were projected on the chamber’s ceiling to counteract any sleepiness.

The water temperature was adjusted by a temperature-controlled bath (BQ100, Yamato Scientific Co., Tokyo, Japan). For the separate water circulation of the upper and lower parts of the tube-lined suit, two pumps were used (Delta Wing Pump, Allen-Vanguard Corp.). Three different LBNP conditions were measured in random order in each thermal condition. The inter-test interval was set at 23 min: a 6-min baseline, 12-min LBNP, and 5-min break time (see Figure 2). The water temperature for the water-perfused tube-lined suit for thermoneutral condition was set at 32°C. For the whole-body heating condition, the water temperature was increased to 42°C within 30 min after the thermoneutral condition was confirmed. After the 30-min heating period, three different LBNP conditions during whole-body heating were measured.

### Data acquisition and analysis

Temperature data were recorded by a data logger (LT-8A; Gram Corp., Saitama, Japan) at 2-s intervals. Rectal temperature (Tre) was measured by a thermistor (LT-ST08-11; Gram) with a disposable rubber sheath (RC5020-A; Nikkiso-Therm Co. Tokyo) inserted 12 cm into the rectum. Skin temperatures (Tsk) were also measured using a thermistor (LT-ST08-00; Gram) fixed to the skin with adhesive surgical tape (Surgical Tape-21 N; Nichiban Co. Tokyo, Japan). Skin temperatures were measured at the forehead (Tsk-head), abdomen (Tsk-abdomen), and dorsum of the left foot (Tsk-foot). These three skin-temperature measuring points were not covered by the water-perfused tube-lined suit. The weighting coefficient for the mean skin temperature from these three points was not clear, and therefore the mean skin temperature was not calculated. The water temperature of the temperature-controlled bath and the air temperature in the LBNP chamber were also measured using a thermistor (LT-ST08-00; Gram). The water temperature was 31.96°C ± 0.01°C (SE) (range, 31.93°C to 32.01°C) during the thermoneutral condition, and 41.88°C ± 0.06°C (SE) (range, 41.25°C to 42.03°C) during the whole-body heating condition.

ECG, impedance-cardiogram (ICG), and phonocardiogram (PCG) signals were taken using a polygraph system (AB-621 G for ECG, AI-601 G and ED-601 G for ICG, and AS-601H for PCG; Nihon Kohden Corp., Tokyo, Japan) and were recorded using a personal computer with an analog-to-digital conversion rate of 1 kHz per channel by a 16-bit AD converter (AD16-16U(PCI)EH; Contec Co., Osaka, Japan). From the ICG signal, the basal thoracic impedance (Z_0_), the delta impedance waveform (ΔZ), and its first derivative (dZ/dt) were derived. Z_0_ is inversely related to central blood volume [24]. SV values were estimated by Kubicek’s method using the dZ/dt signal [25]. Blood resistivity (ρ) was set at 135 cm · ohm. To estimate the SV on a beat-by-beat basis, the noise of the dZ/dt signals was eliminated by an adaptive filter that can select the components that are synchronous with the R-R interval (RRI) of the ECG [26]. The same filter was applied to derive the respiration curve from ΔZ [27]. The beat-by-beat heart rate (HR) was calculated from the RRI sequences of the ECG. CO was also calculated (= SV × HR) on a beat-by-beat basis. AP was measured intermittently by a non-invasive oscillometric blood pressure monitor (HEM-7200; Omron Healthcare, Kyoto, Japan). AP was not measured continuously because of the limitations of our experimental facility. The measuring interval was set at approximately 100 s to avoid the biased distribution of the measuring timing in the sinusoidal phase of LBNP. Therefore, at each measuring interval, the lag angle against the sinusoidal LBNP for the start timing of cuff inflation was shifted 120° and 200° for the 30-s and 180-s conditions, respectively. The AP value was averaged for the baseline and for the LBNP period. The TPR was calculated by dividing the mean arterial pressure (MAP) by the CO. Therefore, the periodic responses of MAP and TPR cannot be described in this study.

The first 3 min of data in the LBNP period were not used for the analysis, to ensure the stabilization of the physiological condition of the subject. The last 3 min of data of in the LBNP period were also eliminated from the analysis, to align the same data length to 6-min baseline period data. The unequal intervals of the beat-by-beat data of RRI, SV, and CO were interpolated into 5.689-Hz (= 1,024 point/180 s) equidistant data. The 6-min RRI dataset, with the Hanning window after linear trends were eliminated by linear regression, was used for the HRV analysis. The coarse graining spectral analysis (CGSA), the algorithm developed by Yamamoto and Hughson [28], was applied for the HRV analysis. The advantages of using the CGSA for an HRV analysis were described [29]. The fractal-free high-frequency (HF) and low-frequency (LF) components were integrated from 0.15 to 0.50 Hz and from 0.05 to 0.15 Hz, respectively, of the power spectra. The HF components of HRV are considered markers of cardiac vagal activity, whereas the LF components are markers of both cardiac vagal and sympathetic activities [30]. The LF/HF ratio was calculated for the index of sympathovagal balance [31].

To normalize the distribution of HRV parameters, we used the natural logarithmic transformed values for the analysis (that is, the natural logarithmic of the LF component of HRV (lnLF), the natural logarithmic of the HF component of HRV (lnHF), and the natural logarithmic ratio of LF/HF of HRV, or ‘ln(LF/HF)’). The advantages of the natural logarithmic transformation in HRV were thoroughly explained in analyses of the data of relatively large numbers of subjects [32]. To check the contamination of the respiratory component in the LF component of HRV, we also calculated the percent LF to total power (TP; 0.00-0.50 Hz) of the respiration curve from ΔZ.

The transfer function data between the cardiovascular indices (RRI, SV, CO, and Z_0_) and the sinusoidal gauge pressure of LBNP were estimated from the same time period of the dataset of the HRV analysis. Gain, phase, and coherence during the LBNP period were derived from the cross-spectral analysis on a fast Fourier transformation basis [11]. These transfer function data were analyzed only during sinusoidal LBNP. The baseline period and the constant LBNP condition were excluded from the transfer function analysis.

A questionnaire regarding lifestyle habits was completed by each subject. The questionnaire gathered information about the subject’s habitual physical exercise per week, average exposure time to air-conditioned rooms per day, and the frequency of going without breakfast [33]. The Japanese version of the Morningness-Eveningness Questionnaire (MEQ) score by Horne and Östberg [34] regarding chronobiological rhythms was also completed by each subject.

### Statistical analysis

The effects of heat stress (‘Heating’, or thermoneutral and whole-body heating) on the mean values during the baseline were analyzed with a repeated-measures analysis of variance (ANOVA). To check the order effect in each thermal condition as a confounding factor on baseline values, three conditions of LBNP order (‘Order’: LBNP1, LBNP2, and LBNP3) and three conditions of LBNP (‘Period’: 30-s period, 180-s period, and constant) were also included in the factors of ANOVA. The difference of mean values from baseline and LBNP were analyzed with a two-way ANOVA followed by a simple effect test with Bonferroni correction for the *post-hoc* analysis (SPSS Inc., Chicago, IL, USA). The factors were two levels of heat stress (Heating) and three levels of LBNP condition (Period). For the transfer function data of gain, phase, and coherence, the effects of heat stress (‘Heating’: thermoneutral and whole-body heating) and two conditions of LBNP (‘Period’: 30-s period and 180-s period) were analyzed by a two-way repeated-measures ANOVA followed by a simple effect test with Bonferroni correction for the *post-hoc* analysis (SPSS). The units of the phase (degree) and coherence (coefficient) were identical in the cardiovascular variables. The comparison of variables was performed with a repeated-measures ANOVA. The Greenhouse-Geisser correction was used to evaluate *P* values for repeated measures involving more than one degree of freedom. Greenhouse-Geisser *P* values were based on corrected degrees of freedom, but the original degrees of freedom are reported. A linear regression analysis was used to examine the relationship between the cardiovascular index and the results of the lifestyle habits questionnaire. To analyze differences in the regression coefficients (that is, the slope of the regression line) among the heating conditions, we used an analysis of covariance (ANCOVA) [35]. All data are expressed as mean ± SE, and the level of significance was set at *P* < 0.05.

## Results

### Physical data

The average gauge pressure (CLC) of LBNP was set at −25 mmHg in all conditions. There were marginal but significant differences in the gauge pressure among the LBNP conditions (30-s period, -24.65 ± 0.06 mmHg; 180-s period, -24.92 ± 0.08 mmHg; constant, -25.05 ± 0.03 mmHg); *F*(2, 22) = 15.08, *P* <0.001. The differences of 0.40 mmHg (30-s *vs.* constant) and 0.27 mmHg (30-s *vs.* 180-s) were both significant.

The air temperature in the LBNP chamber was significantly affected by heating condition (thermoneutral, 30.00 ± 0.10°C; whole-body heating, 32.15 ± 0.07°C; *F*(1,11) = 755.22, *P* <0.001) and by LBNP (baseline, 31.53 ± 0.10°C; LBNP, 30.62 ± 0.07°C; *F*(1,11) = 209.94, *P* <0.001). The heating-condition effect was caused mainly by the convection and the radiation of heat from the water-perfused tube-lined suit, and the LBNP effect was caused mainly by Charles’s law.

### Baseline data

The results of baseline values under the thermoneutral and heating conditions are shown in Table 1. Whole-body heating significantly increased the baseline values of Tre (*P* <0.05), Tsk-abdomen (*P* <0.001), Tsk-foot (*P* <0.05), HR (*P* <0.05), TPR (*P* <0.05), and ln(LF/HF) of HRV (*P* <0.01), but decreased the SV (*P* <0.01), CO (*P* <0.05) and lnHF of HRV (*P* <0.01). As for the order effect (Order), there was a significant interaction between Order and Heating condition at baseline in Tre (thermoneutral, -0.12 ± 0.02°C (LBNP1 *vs.* LBNP3) and whole-body heating, +0.14 ± 0.03°C (LBNP1 *vs.* LBNP3); *F*(2,22) = 47.37, *P* <0.001. However, the main effect of LBNP condition (Period) and the interaction between Period and Heating condition were not significant in the baseline values of all variables. Therefore, the order effect as the confounding factor was randomized and minimized among the LBNP conditions.

**Table 1:** Mean values of baseline under the thermoneutral and heating condition, and the result of ANOVA

	**Theremoneutral**	**Heating**	**Result of ANOVA F**_**(1,11)**_**=**
Tre (°C)	37.15 (0.10)	37.25 (0.08)	5.05^a^
Tsk-head (°C)	35.08 (0.10)	35.03 (0.08)	0.27
Tsk-abdomen (°C)	35.43 (0.16)	36.42 (0.10)	83.19^b^
Tsk-foot (°C)	33.70 (0.32)	34.53 (0.16)	7.51 ^a^
HR (bpm)	63.25 (2.31)	66.32 (2.21)	8.66 ^a^
SV (mL/beat)	96.17 (6.14)	87.37 (5.31)	13.92^c^
CO (L/min)	5.96 (0.31)	5.70 (0.28)	6.89 ^a^
Z_0_ (ohm)	25.87 (0.53)	25.79 (0.52)	0.56
MAP (mmHg)	80.11 (1.95)	80.94 (2.51)	0.98
TPR (mmHg/(l/min))	13.78 (0.75)	14.52 (0.80)	9.02 ^a^
ln(LF) (ln(msec^2^))	4.51 (0.28)	4.55 (0.21)	0.04
ln(HF) (ln(msec^2^))	5.16 (0.27)	4.69 (0.30)	15.37 ^c^
ln(LF/HF) (ln(ratio))	−0.65 (0.35)	−0.14 (0.28)	11.95 ^c^

Mean(SE), *n* = 12, ^a^*P* <0.05; ^b^*P* <0.001;^c^*P* <0.01.

### Response to LBNP of hemodynamic data

**Figure 3:**
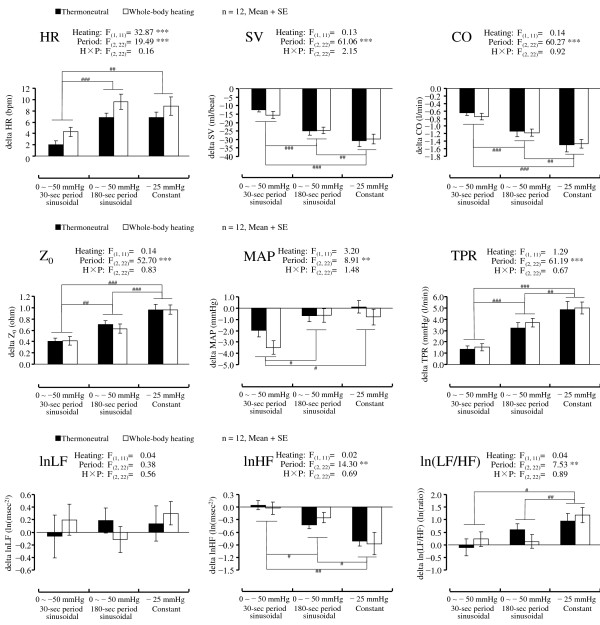
**Differences in mean values (+SE) from baseline to LBNP in HR, SV, CO, Z_**0**_, MAP, TPR, lnLF, lnHF, and ln(LF/HF) of HRV.** ***P <0.001, **P <0.01, *P <0.05 significant difference in ANOVA. ###*P* <0.001, ##*P* <0.01, #*P* <0.05 significant difference in simple main effect of period condition. in each heating condition.

The differences in mean values from baseline to LBNP in each condition are shown in Figure 3. The main effect of the Heating was significant only for HR (*P* <0.001). Whole-body heating augmented the increase in HR during LBNP. There were significant main effects of Period in HR (*P* <0.001), SV (*P* <0.001), CO (*P* <0.001), Z_0_ (*P* <0.001), MAP (*P* <0.01), and TPR (*P* <0.001). The simple main effect of the Period condition showed that the effect of LBNP was unique in MAP. The other variables showed a relatively small variation in the 30-s period condition compared to the 180-s period and constant LBNP conditions, whereas the MAP results showed a large drop in the 30-s period condition.

### Response of the HRV data to LBNP

The contamination of the respiratory component in the LF band was negligible. The ratio of LF band power to TP of the respiration curve was 1.46 ± 0.39%. The spectral leakages of gauge pressure of sinusoidal LBNP in the LF and HF bands were also negligible. The ratios of LF and HF to TP were 0.022 ± 0.005% (LF band) and 0.010 ± 0.002% (HF band), respectively.

Figure 3 shows the differences in mean values from baseline to LBNP of the lnLF, lnHF, and ln(LF/HF). There were significant main effects of Period in lnHF (*P* <0.01) and ln(LF/HF) (*P* <0.01). The simple main effect of the Period condition showed that the effect of LBNP was relatively large in the constant LBNP condition in both lnHF and ln(LF/HF).

### Transfer function data

**Figure 4:**
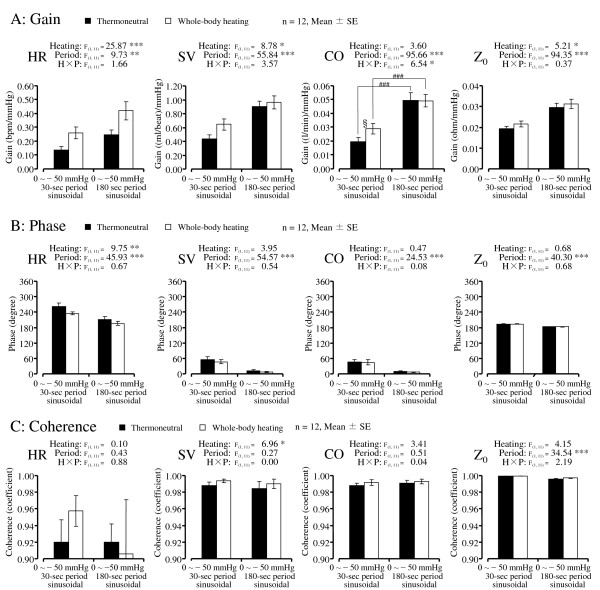
**Results of transfer function data between LBNP gauge pressure (input signal) and HR, SV, CO, and Z_**0**_ (output signal).** Gain (row **A**), phase (row **B**), and coherence (row **C**) in the LBNP period. ***P <0.001, **P <0.01, *P <0.05 significant difference in ANOVA. §*P* <0.05 significant difference in heating conditions in each period. ###*P* <0.001 significant difference in period conditions in each heating condition.

Figure 4 shows the results of transfer function data between LBNP gauge pressure and HR, SV, CO, and Z_0_ of gain (Figure 4, row A), phase (row B), and coherence (row C) in the LBNP period and the result of the repeated-measures two-way ANOVA.

Regarding the transfer gain results, the main effect of the Heating was significant in HR (*P* <0.001), SV (*P* <0.05), and Z_0_ (*P* <0.05). The gain reflects the amplitude of the oscillation at the LBNP period. In the whole-body heating condition, large amplitudes of HR, SV, and Z_0_ were observed. The main effect of the Period was significant in HR (*P* <0.01), SV (*P* <0.001), CO (*P* <0.001), and Z_0_ (*P* <0.001) with relative small variation in the 30-s period condition compared to the 180-s period. There was a significant interaction between the Heating and Period in CO (*P* <0.05). The simple main effect showed that the effect of the Heating was significant in the 30-s period condition (*P* <0.05) but not in the 180-s period condition.

Regarding the transfer phase results, the values of HR and Z_0_ indicated relative large phase angles compared to SV and CO. These large difference between the parameters were reflecting the opposite response direction to LBNP. The HR and Z_0_ increased during LBNP, but the SV and CO decreased (Figures 1, 3). There was a significant main effect of the Heating in HR. The positive phase angle reflects the delay of the oscillation to the sinusoidal LBNP. Whole-body heating decreased the lag angle of HR. However, the main effect of Period was significant in all variables (HR, SV, CO, and Z_0_, *P* <0.001 for all variables). The lag angle to the sinusoidal LBNP was relatively large in the 30-s period condition. The comparison of variables revealed that phase was significantly different between the variables (*F*(3,33) = 686.04, *P* <0.001). The simple main effect showed that the lag angle in HR (*P* <0.01) was relatively large compared to that in Z_0_.

Regarding the transfer coherence results, the main effect of Heating was significant in SV (*P* <0.05). The coherence reflects the similarity of the oscillation to the sinusoidal LBNP. Under the whole-body heating condition, relatively large coherence was shown in SV. The main effect of Period was significant in Z_0_ (*P* <0.001) with relatively small coherence in the 180-s period condition compared to the 30-s period.

### Linear regression analysis

**Figure 5:**
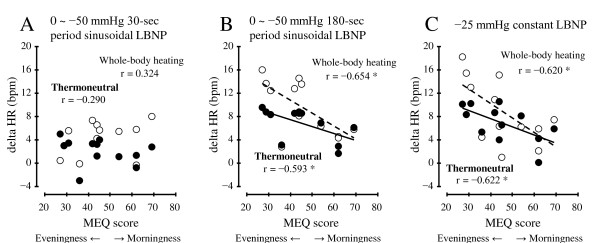
**Linear regression analysis of the inter-individual relationship between the morningness-eveningness questionnaire (MEQ) score and the difference in mean values from baseline to LBNP in HR during thermoneutral (*filled circle*, solid line) and whole-body heating (*open circle*, dashed line).** *P <0.05. *n* = 12.

The linear regression analysis examining the cardiovascular index and the results of the lifestyle habits questionnaire showed that the correlation of HR and MEQ score was significant. The habitual physical exercise times per week, the average exposure time to an air-conditioned room per day, and the frequency of going without breakfast were not significantly correlated with the cardiovascular index. Moreover, the significant correlation between HR and MEQ score was shown in the 180-s period and the constant LBNP condition (*P* <0.05 for both conditions), but not in the 30-s period LBNP condition (Figure 5). The subjects with higher Morningness scores tended to adjust to the 180-s period and constant LBNP by a small increase in HR. The correlation coefficient between start time of measurement and MEQ score was not significant (*r* = −0.174, *P* = 0.588, n.s.). When the ANCOVA was performed on HR and MEQ score, there were no significant differences in regression coefficients between the Heating conditions in the 180-s period (*F*(1,20) = 0.60, n.s.) and the constant LBNP condition (*F*(1,20) = 0.46, n.s.).

## Discussion

The major finding of this study was that the cardiovascular responses, except the MAP, were attenuated in the sinusoidal (PLC plus CLC) LBNP compared to the constant (CLC only) LBNP, although the average gauge pressure (CLC) during the LBNP period was set at −25 mmHg in all conditions. The mean Z_0_ values showed that the degree of the thoracic blood volume shift to LBNP was the largest in the constant LBNP condition, second largest in the 180-s period condition, and the smallest in the 30-s period condition (Figure 3). Z_0_ is inversely related to central blood volume [24]. The order of the degree of the variation against each LBNP condition was the same in the decreases of the SV and CO (Figure 3) and in the rise of the TPR (Figure 3). As for the orthostatic regulation of blood pressure, the MAP results showed a large drop in the 30-s period condition (Figure 3), whereas the other cardiovascular indices showed relative small variation in that condition. These results confirmed the previous report [13] that the cardiovascular adjustability to sinusoidal LBNP was maintained at the period of slower than 50-s (that is, 0.02 Hz) oscillation. Levenhagen *et al*. (1994) revealed that cardiovascular adjustability to sinusoidal LBNP was maintained >50 s of the period of oscillation with an amplitude of 25 mmHg of LBNP. The amplitude of 25 mmHg of sinusoidal LBNP has a CLC of −25 mmHg. However, to our knowledge there is no previous study that compares sinusoidal LBNP with constant LBNP. The results of the present study revealed that the effect of the CLC of LBNP on cardiovascular adjustability was attenuated by the addition of PLC to LBNP.

Considering that the spectral leakages from the main periodic component of oscillatory LBNP directly contaminate the other spectral components [11], the waveform of LBNP should be a sinusoidal pattern in cases of simultaneous measurements of HRV during oscillatory LBNP. In the present study’s HRV results, the response of lnHF and ln(LF/HF) to LBNP were attenuated in the sinusoidal LBNP compared to the constant LBNP. Since there was no significant effect on lnLF, the ln(LF/HF) results reflect a dominant effect of lnHF, which is an index of vagal activity [30]. Therefore, we surmise that the attenuated cardiovascular adjustability by the addition of periodic oscillation of LBNP was caused by the suppression of the vagal responsiveness to LBNP.

Regarding the whole-body heating, the baseline of Tre was raised by almost 0.1°C by this experimental protocol. Previous studies showed the increase of their subjects’ core temperature from 0.5°C to 1.5°C by a water perfusion suit with a relatively higher water temperature [10,15]. Although the heating condition in the present study was very mild whole-body heating, the significant main effect of the Heating on baseline values was observed for Tre, Tsk-abdomen, Tsk-foot, HR, SV, CO, TPR, lnHF, and ln(LF/HF). We suspect that the significant main effect of the Heating on ln(LF/HF) is the result of relative sympathetic activation during whole-body heating. However, there was no significant effect on lnLF. Moreover, there is an argument that the LF of HRV is not a biomarker of sympathetic activity but rather is a measure of the modulation of cardiac autonomic outflows by the baroreflex [36]. Generally, heat stress does not alter the baroreflex control of the heart [37], and therefore, the present study’s finding on lnLF could be adequate. The baseline values of lnHF and SV dropped significantly during the Heating condition. The decreases in HF of HRV and SV are known to indicate a reduction in venous return [38]. However, since the CO baseline values were decreased by the heating although MAP was maintained in our study, TPR was estimated that was increased in the heating condition. In previous studies using whole-body heating, the TPR dropped markedly with heating [39]. However, heat stress induces vasoconstriction in non-cutaneous beds (that is, splanchnic, renal, muscle, and cerebral) [14,40]. The present finding of a relatively small increase in TPR might reflect non-cutaneous vasoconstrictions caused by the very mild whole-body heating. We should have measured cutaneous vascular resistance (CVR) in reference to the TPR.

As for the mean values’ responses to LBNP, the ANOVA showed that the main effect of Heating was significant only in HR (Figure 3). However, in the gain of the transfer function, the significant main effect of the Heating was observed in HR, SV, and Z_0_ (Figure 4). As for the influence of temperature on the distribution of blood, which was examined by right heart catheterization [41], the measurement of Z_0_[42], and gamma camera imaging [43], heat stress induces a central blood volume reduction. Although the heating conditions used in the present study were very mild compared to those of previous studies, we were able to detect the significant increase in the gain of Z_0_ in the Heating condition. Considering that the beat-by-beat signal of the cardiovascular response contains other periodic fluctuations (that is, respiratory sinus arrhythmia and Mayer wave-related sinus arrhythmia), a transfer function analysis of a target sinusoidal period of LBNP would be advantageous to experimentally detect cardiovascular adjustability, with high repeatability.

Regarding the phase results and the transfer function, a significant main effect of the Period was observed in all variables (HR, SV, Co, and Z_0_; see Figure 4). These results confirmed those of [13], who showed the relatively large lag angle to the sinusoidal LBNP in a short period of condition. The phase angle of HR and Z_0_ were relatively large compared to SV and CO, reflecting the adverse response direction to LBNP. Within the variables of the same response direction to LBNP, the lag angle in HR was relatively large compared to Z_0_, reflecting the cascade reaction to LBNP (that is, the reduction of central blood volume could be preceding the HR response to LBNP). Moreover, the significant main effect of the Heating on the HR of phase of the transfer function indicated that the lag angle in the HR was decreased by heat stress. Considering the augmented gain in HR caused by whole-body heating, the HR response to LBNP in heat stress was characterized by a large amplitude and quick reaction. In this study, the AP was not measured continuously because of the limitations of our experimental facility. The dynamic responses of AP, TPR, and also CVR to sinusoidal LBNP should be investigated to increase our understanding of orthostatic intolerance under heat stress.

Our finding of distinctly high coherence of Z_0_ reflected the similarity to the sinusoidal LBNP. Z_0_ is inversely related to central blood volume [24]. The similarity was close to a coefficient of 1.0 (Figure 4). The main effect of Period on the Z_0_ of phase indicated that the coherence in Z_0_ was relatively lower in the 180-s period condition compared to the 30-s period. This might not have been caused as a physiological consequence, because the set value of the 180-s period of sinusoidal LBNP curve has a relatively long duration to maintain above −0.6 mmHg, which was the upper limit of the controllable range of gauge pressure of the LBNP system we used. Nevertheless, sinusoidal LBNP caused a sinusoidal thoracic blood shift.

As for the association between the subjects’ lifestyle habits and their cardiovascular responses to LBNP, the linear regression analysis showed a significant correlation between HR and MEQ score, except for the 30-s period LBNP condition. The subjects’ dietary habits and daily physical activity were not significantly correlated with their cardiovascular responses to LBNP. Previous studies showed that individual differences in MEQ score were related to the subjects’ baseline HR [21], sleeping behavior [44], personality [45], and mental health [46]. The previously reported diurnal variation in vascular function indicates that the vasoconstrictor response is lower in the morning than afternoon [47,48]. The reduced vascular function could cause the higher cardiac responsiveness to maintain the AP against the LBNP [49]. Although high HR responsiveness is not directly correlated with LBNP tolerance [1], we suspect, but have not proved, that the reduced physiological arousal in evening-type subjects might contribute to the high HR responsiveness to the LBNP. A previous study reported that higher HR values with a low MEQ score (Evening type) was associated with low vagal activity [21].

The present study has several limitations. There were marginal but significant differences in the gauge pressure between the LBNP conditions. The differences of 0.40 mmHg (30-s *vs.* constant) and 0.27 mmHg (30-s *vs.* 180-s) were both significant. The magnitude of LBNP is directly related to the reductions of CVP [2,13]. These data suggest 2-mmHg decreases of CVP for every −10 mmHg LBNP. The physiological significance of the difference of 0.40 mmHg of LBNP is not known, but it might be negligible. These marginal but significant differences may have been caused by the combination of accurate repeatability of the sinusoidal LBNP and the mismatch of PID parameters in the LBNP control system. This issue merits further study.

## Conclusions

In conclusion, the effect of the CLC of LBNP on cardiovascular adjustability was attenuated by the addition of the PLC of LBNP. In light of the results of the simultaneous measurements of HRV, we suggest that the attenuation may be caused by the suppression of the vagal responsiveness to LBNP. We observed that the short period of PLC decreased the response in HR, SV, CO, and TPR, but increased the response in MAP under the same CLC condition. Moreover, these PLC effects were augmented by very mild whole-body heating. Further studies to investigate the association between the broad range of lifestyle habits and the large inter-individual variation in orthostatic tolerance under heat stress are warranted.

## Abbreviations

ANCOVA: Analysis of covariance; ANOVA: Analysis of variance; AP: Arterial pressure; CGSA: Coarse graining spectral analysis; CO: Cardiac output; CVP: Central venous pressure; CVR: Cutaneous vascular resistance; CBF: Cerebral blood flow; HF: High frequency; HR: Heart rate; HRV: Heart rate variability; ICG: Impedance-cardiogram; LBNP: Lower body negative pressure; LF: Low frequency; lnLF: Natural logarithmic of LF component of HRV; lnHF: Natural logarithmic of the HF component of HRV; ln(LF/HF): Natural logarithmic ratio of LF/HF; MAP: Mean arterial pressure; MEQ: Morningness-Eveningness Questionnaire; PCG: phonocardiogram; PID: Proportional-integral-derivative; RRI: R-R interval; SV: Stroke volume; TP: Total power; TPR: Total peripheral resistance; Tre: Rectal temperature; Tsk-head: Skin temperature at the forehead; Tsk-abdomen: Skin temperature at the abdomen; Tsk-foot: Skin temperature at the dorsum of the left foot; Z_0_: Basal thoracic impedance; ΔZ: Delta impedance waveform; dZ/dt: First derivative of ΔZ.

## Competing interests

The authors have no competing interests to disclose.

## Authors’ contributions

KI collected data and wrote the manuscript; MT and SH edited the manuscript and did the data interpretation; KI and AY revised the manuscript critically for important intellectual content. All authors read and approved the final manuscript.
